# Diagnosis of Benign Paroxysmal Positional Vertigo: Review of Different Diagnostic Methods

**DOI:** 10.3390/audiolres16050137

**Published:** 2026-09-17

**Authors:** Elena A. Shestel, Dmitry V. Burtsev, Pavel S. Zubanov, Arcadiy S. Goldberg, Pavel P. Tregub

**Affiliations:** 1 Regional Consultative and Diagnostic Center, Rostov-on-Don 344000, Russia; eashestel@live.ru (E.A.S.); omldc@omldc-rnd.ru (D.V.B.); 2 Central Research Institute of Epidemiology, Moscow 111123, Russia; tregub@cmd.su; 3 Semashko National Research Institute of Public Health, Moscow 105064, Russia; goldarcadiy@gmail.com; 4 Sechenov First Moscow State Medical University, Moscow 119991, Russia; 5 Research Center of Neurology and Neurosciences, Moscow 125310, Russia

**Keywords:** benign paroxysmal positional vertigo, BPPV diagnostic methods, videonystagmography, vertigo

## Abstract

Benign paroxysmal positional vertigo (BPPV) is the most common cause of patient-reported vertigo. The accurate diagnosis of BPPV requires high-quality physician training and knowledge of the advantages and disadvantages of different diagnostic methods. Most studies focus on a limited number of diagnostic approaches; the purpose of this review is to consolidate and systematize the available information on the wide range of existing methods. This review includes comparative descriptions of the clinical utility of 12 different techniques used to diagnose BPPV. The diagnosis of BPPV relies primarily on a thorough analysis of clinical history and the professional application of positional tests (primarily the Dix–Hallpike and the Pagnini–McClure maneuvers). Several methods can be used to enhance the efficiency of positional testing: rotary chair, electronystagmography (ENG), videonystagmography (VNG), and potentially artificial neural networks for the analysis of VNG results. The VEMP (vestibular evoked myogenic potential) technique, vestibular caloric testing, the video head impulse test, computerized posturography, and CT and MRI of the head are all useful auxiliary methods for the differential diagnosis of BPPV in complex cases. When selecting diagnostic approaches, it is important to consider the balance between resource and time expenditure and diagnostic accuracy. This review enables a comprehensive and well-rounded understanding of a wide range of diagnostic methods, which is necessary for the development of optimal diagnostic and subsequent treatment and monitoring tactics.

## 1. Introduction

Benign paroxysmal positional vertigo (BPPV) is the most common cause of patient-reported complaints of vertigo, accounting for 20% to 35% of all patients that present with dizziness to physicians of various specialties and more than half of dizziness cases not associated with central nervous system disorders [1,2,3]. Despite the benign course of this condition, it can significantly reduce patients’ quality of life, as they are often forced to reduce their level of daily activity and face limitations in sports and professional tasks [4].

All epidemiological studies on BPPV encounter the issue that medical records are not entirely accurate; diagnostic errors in BPPV cases occur quite frequently, and one of the main reasons for such errors is the extensive list of conditions requiring differential diagnosis [5,6]. For example, these conditions include Ménière’s disease, labyrinthitis or vestibular neuritis, ossifying labyrinthitis [7,8], post-traumatic dizziness, perilymphatic fistula, inner ear injuries [9,10], manifestations of migraine, transient cerebrovascular accidents, demyelinating diseases (e.g., multiple sclerosis), vertebrobasilar insufficiency, central positional vertigo due to other causes of brain damage [11], anxiety disorders, cervicogenic dizziness [12], vestibular migraine, alcohol intoxication, orthostatic hypotension [13,14], vascular loop syndrome, and the use of medications with potential ototoxic effects [15,16].

Patients suffering from dizziness may present to physicians in different specialties, such as neurologists, otorhinolaryngologists, and general practitioners. The accurate diagnosis in such circumstances requires attentiveness, high-quality professional training, and knowledge of the diagnostic methods for BPPV, including their advantages and disadvantages.

Most of the scientific literature focuses on a limited number of diagnostic methods; the purpose of this review is to consolidate and systematize the available information on the wide range of existing techniques. This review includes comparative descriptions of the clinical utility of 12 different diagnostic methods used to diagnose BPPV, including sensitivity data and the advantages and possible problems associated with these approaches. This work contributes to a comprehensive understanding, which is necessary for the development of optimal diagnostic and subsequent treatment and monitoring tactics.

## 2. Methodology

Our literature search was conducted in open and subscription-based electronic databases: PubMed/MEDLINE, Scopus, and Google Scholar. The search period spanned from 1990 to 2025.

Inclusion criteria for the review included the following: original clinical studies (cohort, cross-sectional, controlled); systematic reviews and meta-analyses; clinical guidelines and recommendations; and studies containing quantitative data on diagnostic accuracy (sensitivity, specificity) of methods used for BPPV diagnosis.

Exclusion criteria included the following: individual case reports; studies in which BPPV diagnosis was not the primary focus of the research; conference abstracts and non-peer-reviewed publications; and articles that did not contain data on the clinical utility of diagnostic methods or that exclusively described therapeutic approaches.

Data on the sensitivity, specificity, reproducibility, limitations, and practical applicability of each diagnostic method were extracted from the selected sources. A total of 103 key sources were selected for citation.

## 3. Clinical History Analysis for BPPV Diagnosis

Patients suffering from BPPV typically share a number of characteristic features. Patients describe short, recurrent episodes of dizziness provoked by changes in their head position relative to gravity, often perceived as a systemic sensation of spatial rotation (“riding a merry-go-round”). Common triggers include turning over in bed, looking upward, or bending forward. These episodes are typically brief, lasting less than 1 min. Some individuals may report nonspecific dizziness, presyncopal sensations, nausea, or balance disturbances. Between episodes, the condition may be asymptomatic; however, up to 50% of patients may experience sensations of unsteadiness or balance disturbance even outside of an attack. Symptoms are paroxysmal and stereotypical in nature, and patients sometimes modify their movements to avoid triggering episodes [17,18].

BPPV is more commonly diagnosed in older patients and women. In elderly individuals, age-related degeneration of the otolithic membrane is more likely, which can lead to the development of BPPV. However, many conditions requiring differential diagnosis (the list of which is provided above) are also more likely to manifest in elderly patients [19,20].

Distinguishing between vestibular pathologies and causes related to the central nervous system is critical. To identify these differences, it is essential to obtain the most detailed description of symptoms and ascertain the time of their onset, contextual factors, and factors that exacerbate and/or alleviate symptoms. Additionally, physicians should obtain information regarding recent viral infections due to their possible association with labyrinthitis, history of trauma, recent neurosurgical interventions, and medication use [16,21].

Overall, more than 50% of BPPV cases are idiopathic—i.e., they occur without any identifiable specific cause [22]. Numerous studies have been published on risk factors for BPPV development, and attention should be paid to the presence of these factors during history collection and analysis. While some data are contradictory, many factors demonstrate a convincing, generally recognized correlation, including (in addition to those already mentioned above) genetic predisposition (family history of BPPV) [23], strong acoustic or electrical stimulation [24,25], temporal bone trauma and surgery [26,27], osteoporosis or low bone mineral density, vitamin D deficiency in postmenopausal women [28,29], diabetes mellitus [30], hyperlipidemia, thyroid disease [31], migraine, and mood disorders [32,33]. Some of the listed risk factors (for example, injuries) can not only contribute to the development of BPPV but may also (perhaps even more likely) cause vertigo that can be mistaken for BPPV, thereby creating additional difficulties in differential diagnosis. That is why it is especially important to record the presence of these factors during the patient examination [1,24,25].

One possible method of history collection and analysis is the use of patient-completed questionnaires. Several studies have examined the utility of questionnaires for confirming BPPV and determining its subtypes based on characteristics (positional provocation, duration, etc.) of dizziness and positional changes; the proposed questionnaires demonstrate relatively acceptable sensitivity and specificity and may be useful for clinicians [34,35,36]. Some questions in these surveys are designed to identify possible BPPV, while others help determine the subtype of the disease and the affected ear. When patients complete the questionnaire on electronic devices, not on paper, the software can assist both the patient and the physician in interpreting responses [37,38].

In essence, working with electronic questionnaires does not extend beyond routine history collection and analysis, but it presents additional opportunities for the self-diagnosis of BPPV and can also assist physicians lacking sufficient experience and training.

History collection and analysis often allow for suspicion of BPPV with a high degree of probability; however, the diagnostic value of history has limitations. In some cases, particularly in elderly patients, dizziness may be perceived as non-systemic and described indirectly (“queasiness” or “lightheadedness”), and the specific provoking factor may not be recognized by the patient.

Furthermore, at the onset of the disease, autonomic symptoms (nausea, vomiting, tachycardia) may dominate over dizziness. Thus, a diagnosis of BPPV based exclusively on anamnesis does not offer sufficiently high sensitivity and specificity for the final verification of the diagnosis.

## 4. Physical Examination and Positional Tests

The International Classification of Vestibular Disorders (ICVD), developed by the Barany Society, has established diagnostic criteria for BPPV. In addition to the characteristic symptoms described above, which are identified during history collection, an obligatory criterion is characteristic nystagmus induced by a positional maneuver chosen according to the subtype of the disease and the affected ear [39].

The Dix–Hallpike maneuver remains the primary standardized clinical test for the diagnosis of posterior semicircular canal BPPV. In affected patients, this test elicits torsional, upward-beating nystagmus accompanied by dizziness [18,40,41]. During the Dix–Hallpike maneuver, the patient initially sits upright with their head rotated 45 degrees toward the ear being examined. Then, with eyes open, the patient is gently moved to a supine position, with the neck extended 20–30 degrees and the head maintained in the 45-degree rotated position. After 30–60 s, the patient is returned to the sitting position. If nystagmus and dizziness do not occur, the procedure is repeated on the opposite side.

Nystagmus may appear with a latency of 5 to 20 s; its intensity increases and then decreases, and it disappears within 60 s. Nystagmus often changes direction when the patient is returned to the upright position. The intensity of the response to the Dix–Hallpike test varies individually among patients [42]. For patients with horizontal semicircular canal BPPV, the Pagnini–McClure maneuver, also called the roll test, is used. During a head rotation of 90 degrees in the supine position, geotropic or apogeotropic bidirectional nystagmus is detected. Patients’ subjective sensations of dizziness also serve as a useful indicator. Difficulty may arise in determining the side of involvement. Nystagmus may appear upon head rotation for both sides; if BPPV is associated with canalolithiasis, nystagmus will be stronger upon the head rotation toward the affected ear. If the BPPV is caused by cupulolithiasis, nystagmus will be stronger upon the head rotation to the opposite side. The affected ear can be accurately identified in 75% of cases with apogeotropic nystagmus and in 95.6% of cases with geotropic nystagmus [43,44].

In controversial cases, when the affected side cannot be determined with certainty because the intensity of the nystagmus and dizziness is equal upon head rotation to both sides, the clarifying “bow and lean test” can be used. During this test, the patient sits on the examination couch facing the physician and bends their head forward 90° then extends their head backwards 45°. The physician assesses the nystagmus that appears with the head bent forward and the head extended backward. In geotropic nystagmus, the affected side is the one toward which nystagmus occurs upon forward head bending; in apogeotropic nystagmus, it is the side opposite to the direction of nystagmus upon forward head bending [45].

The involvement of the anterior semicircular canal is extremely rare. In such cases, the Dix–Hallpike maneuver described above may be used, as well as the Yacovino test, which involves the deep backward extension of the head without rotation. When downward vertical nystagmus appears, physicians should seek a particularly thorough differential diagnosis considering central vestibular disorders affecting the cerebellum and brainstem [46,47].

Professional use of positional tests is considered the “gold standard” for BPPV diagnosis. The sensitivity of this method is quite high, given its low cost and relative ease of application for qualified specialists. According to various studies, the sensitivity of positional tests ranges from 80 to 95.5%, and specificity is 87.9% [48,49,50]. However, it should be noted that the application of positional tests has several limitations. First, this approach may be difficult to apply in the presence of comorbid conditions (rheumatoid arthritis, kyphoscoliosis, cervical osteochondrosis with limited mobility). Second, in cases of weak or brief nystagmus, a clinical assessment without instrumental enhancement may be insufficiently sensitive, and difficulties may arise in determining the affected side. Third, the characteristics of nystagmus in BPPV (particularly the torsional component) are difficult to visually assess and document [51,52].

In complex cases, a simple tool that can help identify nystagmus, to some extent, is Frenzel goggles. They block the patient’s visual fixation and magnify and illuminate the eyes, enabling the physician to more confidently determine the presence and characteristics of nystagmus [17,53]. Classic optical Frenzel goggles are rarely used today; more commonly used is the modern version of video goggles with infrared cameras, which allow the physician to view the patient’s eyes on a screen. Most BPPV researchers use this tool, as it not only facilitates diagnosis but also allows for reliable result documentation. However, some argue that this method cannot be fully considered an instrumental diagnostic tool, as the positional tests themselves and the signs identified (nystagmus and its characteristics) remain essentially the same and are visually determined by the physician [54,55].

## 5. Instrumental Diagnostic Methods

Rotational diagnostic chairs are a relatively inexpensive method to improve the accuracy and reproducibility of movements that are essentially analogous to the positional tests described above. A number of researchers describe an increase in diagnostic sensitivity when using chairs compared to manual positional testing [56,57,58]. However, in some other studies, rotational diagnostic chairs did not provide an improvement in diagnostic quality for BPPV, or the demonstrated improvement was not sufficiently significant and consistent [59,60].

Both positional tests and rotational chairs are used to check for the appearance of nystagmus. One method that enables objective determination and documentation of nystagmus is electronystagmography (ENG). This approach records nystagmus through electrical potentials captured via electrodes placed around the eyeball. The majority of publications describing the use of ENG date from before 2010; currently, this method is considered relatively obsolete [61,62,63,64].

The disadvantages of the ENG method, which make it inferior to other diagnostic technologies, are primarily related to the accuracy of determining the nature of nystagmus. To accurately distinguish the vertical component of nystagmus from the horizontal position, a simultaneous use of multiple channels is required. The requirement of attaching a high number of electrodes around each eye increases the labor intensity and duration of diagnosis, increasing the likelihood of errors caused by interference due to insufficient electrode–skin contact. During ENG recordings, additional interference is caused by eye blinking and other facial muscle movements [65,66,67,68]. The clinical use of ENG has demonstrated that this method does not always have sufficient sensitivity and cannot reliably and validly record eye movements with an amplitude of less than five degrees. Particular difficulties arise when recording the rotational (torsional) component of nystagmus [69,70,71].

Videonystagmography (VNG) has replaced ENG. VNG is a technological development of classic Frenzel goggles, to which infrared video cameras (recording eye movements in darkness) and information systems have been added to assist the physician in determining the presence and characteristics of nystagmus on video recording. VNG is currently used for the diagnosis and documentation of BPPV signs in most scientific studies [72,73,74].

The VNG method overcomes most of the above-described disadvantages inherent in ENG. VNG has higher sensitivity and can reliably record eye movements with an amplitude of 0.5 angular degrees; it also eliminates sources of interference, such as blinking or poor electrode contact. Instead of attaching numerous electrodes around each eye, a mask is used, which can be quickly and more simply placed on the patient [64,75,76,77]. A particularly interesting direction in the development of VNG technology is the creation and application of portable devices (vestibular event monitors) that patients can carry with them and use during a dizziness attack. This is less useful for diagnosing BPPV and more useful for other pathologies (Ménière’s disease, vestibular migraine); however, the use of wearable devices for the differential diagnosis of BPPV is also possible [78,79].

The evaluation of eye movement video recordings in BPPV is not difficult for an experienced physician. The image of the eye on a screen can be magnified, and the playback speed of the video can be slowed—this makes even the slightest signs of nystagmus noticeable, recorded both by computer software and by the observer, thereby reducing the likelihood of diagnostic errors. It is important not only to detect nystagmus and its characteristics but also to link the moment of its appearance with the positional tests being performed (Dix–Hallpike, Pagnini–McClure, etc.). In recent years, there have been increasing developments utilizing computer neural networks and deep machine learning systems to help physicians consider all factors simultaneously. The application of these technologies makes it possible to create a full-fledged clinical decision support system that can help to reduce the chance of possible diagnostic errors [80,81,82,83].

The technique of vestibular evoked myogenic potentials (VEMPs) in benign paroxysmal positional vertigo remains auxiliary. Depending on what specific muscles’ potentials are recorded, two subtypes of this technique are distinguished: ocular VEMP (oVEMP) and cervical VEMP (cVEMP). oVEMP helps assess the function of the utriculus, which is most often the source of detached crystals [84]. cVEMP assesses the function of the saccule; disorders associated with it are less common in BPPV, but information about them can also be valuable [85]. VEMP is not a primary tool for confirming BPPV but it can provide important additional information in the context of differential diagnosis. This technique may be useful in rare cases, such as complete occlusion of the semicircular canal by an otolithic plug [84,85,86,87].

The vestibular caloric test is another method that is not primarily used to establish a BPPV diagnosis but may be useful for differential diagnosis in some cases. For example, there is convincing evidence that Ménière’s disease can be distinguished from BPPV by parameters determined using caloric testing [88]. Since the application of caloric testing involves the determination of nystagmus under the influence of temperature changes in the external auditory canal, the above-described methods that facilitate nystagmus detection and documentation (ENG, VNG) may be used. One significant limitation of the caloric test is the fact that it can only demonstrate disturbances affecting the horizontal semicircular canals and cannot detect disturbances associated with the anterior and posterior canals [89,90].

The video head impulse test (vHIT) is another auxiliary instrumental technique that is superior to the caloric test in that it enables a sequential assessment of the function of all three pairs of semicircular canals (horizontal, anterior, and posterior). This technique requires special video goggles that not only record the patient’s eye movements but also display a stationary target on which the patient must fixate during head rotations [91,92,93]. The caloric test and the video head impulse test both cannot be used for BPPV primary diagnoses, but they have additional value for the prediction of possible BPPV relapses and for additional monitoring of ongoing treatment [94,95,96].

Computerized posturography is yet another additional instrumental method; it assesses a patient’s ability to maintain balance on a platform equipped with sensors. This method is also supplementary in the management of patients with BPPV and can assist in evaluating the effectiveness of ongoing treatment. Posturography allows for an objective assessment of dizziness manifestations; many elderly patients with BPPV continue to fear vertigo attacks, and their complaints may persist even when treatment is effective. In these cases, posturography is one possible method to evaluate the situation [97,98,99].

Computed tomography (CT) and magnetic resonance imaging (MRI) are valuable tools for the exclusion of infarction, hemorrhage, neoplasms or tumors, and other pathologies that may indicate alternative causes of dizziness. Thus, these methods may be used for differential diagnosis of BPPV [100,101]. However, it should be noted that CT and MRI are methods that require significant time and resource expenditure. They should not be used for the primary diagnosis of BPPV without employing cheaper and more effective diagnostic methods, as this erroneous approach leads not only to increased costs but also to loss of time and diagnostic errors [102,103].

## 6. Conclusions

The diagnosis of BPPV relies primarily on a thorough clinical history analysis and the professional application of positional tests. A number of instrumental methods can enhance the efficiency of positional testing by helping to detect nystagmus and correctly determine its characteristics; among these methods, VNG (videonystagmography) is a modern and accurate method, and additions in the form of artificial neural networks provide hope for a further increase in the efficiency of this approach. Additionally, according to a number of researchers, the accuracy of BPPV diagnosis can be improved through the use of rotational chairs.

The VEMP technique, vestibular caloric test, video head impulse test, computerized posturography, and CT and MRI of the head are all auxiliary methods that are valuable for the differential diagnosis of BPPV in complex and specific cases. To use these methods, additional reasons are needed: additional neurologic symptoms, atypical positional nystagmus or a specific concomitant diagnosis. It can also be noted that the video head impulse test has value for predicting the likelihood of recurrence and assists in monitoring treatment quality.

When selecting diagnostic methods, the balance between resource and time expenditure and diagnostic value should be considered. The general relationship of the costs and diagnostic value for 12 methods is shown in Table 1.

For a small number of complex cases requiring thorough differential diagnosis, patients should be referred to a well-equipped diagnostic center where the necessary diagnostic methods can be applied. At the same time, BPPV is a condition in which more expensive and technologically complex methods are less effective for the primary diagnosis of most cases; the professional training of specialists is much more important. Patients may present with complaints of dizziness to doctors of different specialties (neurologists, general practitioners, otorhinolaryngologists), which means that knowledge of basic diagnostic methods for BPPV should be actively and widely disseminated among medical specialists.

## Figures and Tables

**Table 1:** The relationship between the expenditure of time and resources required for the application of various diagnostic methods (for BPPV) and the diagnostic value of these methods.

Diagnostic Methods		Diagnostic Value of Methods for BPPV	Expenditure of Time and Resources for the Application of Diagnostic Methods	Groups of Diagnostic Methods
1	Analysis of clinical history	High level of diagnostic value	Low level	Basic diagnostic methods for BPPV
2	Positional tests	High level of diagnostic value	Low level	
4	Electronystagmography (ENG)	The method provides additional diagnostic value to the level of positional tests (can be considered obsolete due to known disadvantages)	Middle level	Auxiliary methods that increase the effectiveness of basic positional tests
3	Rotary chair	The method provides additional diagnostic value to the level of positional tests	Low level	
5	Videonystagmography (VNG)		Middle level	
6	VNG with artificial neural network analysis module		Middle level	
7	Vestibular caloric test	Low level of diagnostic value (cannot be used for primary diagnosis but have additional value for predicting possible relapses and monitoring of ongoing treatment)	Low level	Additional instrumental methods for differential diagnosis
9	Video head impulse test (vHIT)		Middle level	
8	Vestibular evoked myogenic potentials (VEMPs)	Low level of diagnostic value (but have additional value for differential diagnosis in some complicated or specific cases)	Middle level	
10	Computerized posturography		High level	
11	Computed tomography (CT)		High level	
12	Magnetic resonance imaging (MRI)		High level	

## Data Availability

No new data were created or analyzed in this study. Data sharing is not applicable to this article.

## Abbreviations

The following abbreviations are used in this manuscript:

BPPV	benign paroxysmal positional vertigo
VNG	videonystagmography
ENG	electronystagmography
VEMP	vestibular evoked myogenic potentials
vHIT	video head impulse test
CT	computed tomography
MRI	magnetic resonance imaging

## References

[1] Koshi E.J., Sutton A.E. (2026). Benign Paroxysmal Positional Vertigo. StatPearls [Internet].

[2] Kerber K.A., Newman-Toker D.E. (2015). Misdiagnosing Dizzy Patients: Common Pitfalls in Clinical Practice. Neurol. Clin..

[3] Grill E., Strupp M., Müller M., Jahn K. (2014). Health services utilization of patients with vertigo in primary care: A retrospective cohort study. J. Neurol..

[4] Lopez-Escamez J., Gamiz M., Fernandez-Perez A., Gomez-Fiñana M. (2005). Long-term outcome and health-related quality of life in benign paroxysmal positional vertigo. Eur. Arch. Otorhinolaryngol..

[5] Nuti D., Masini M., Mandalà M. (2016). Benign paroxysmal positional vertigo and its variants. Handb. Clin. Neurol..

[6] Li S., Wang Z., Liu Y., Cao J., Zheng H., Jing Y., Han L., Ma X., Xia R., Yu L. (2022). Risk Factors for the Recurrence of Benign Paroxysmal Positional Vertigo: A Systematic Review and Meta-Analysis. Ear Nose Throat J..

[7] Kaya S., Paparella M.M., Cureoglu S. (2016). Pathologic Findings of the Cochlea in Labyrinthitis Ossificans Associated with the Round Window Membrane. Otolaryngol. Head Neck Surg..

[8] Caruso G., Nuti D. (2005). Epidemiological Data from 2270 PPV Patients. Audiol. Med..

[9] Choi M.S., Shin S.O., Yeon J.Y., Choi Y.S., Kim J., Park S.K. (2013). Clinical characteristics of labyrinthine concussion. Korean J. Audiol..

[10] Fife T.D., von Brevern M. (2015). Benign Paroxysmal Positional Vertigo in the Acute Care Setting. Neurol. Clin..

[11] Renga V. (2019). Clinical Evaluation of Patients with Vestibular Dysfunction. Neurol. Res. Int..

[12] Cherchi M., DiLiberto F.E., Yacovino D.A., Das S. (2021). The Enduring Controversy of Cervicogenic Vertigo, and Its Place among Positional Vertigo Syndromes. Audiol. Res..

[13] Parnes L.S., Agrawal S.K., Atlas J. (2003). Diagnosis and management of benign paroxysmal positional vertigo (BPPV). Can. Med. Assoc. J..

[14] Swartz R., Longwell P. (2005). Treatment of vertigo. Am. Fam. Physician.

[15] Yetiser S. (2020). Review of the pathology underlying benign paroxysmal positional vertigo. J. Int. Med. Res..

[16] Andersson H., Jablonski G.E., Nordahl S.H.G., Nordfalk K., Helseth E., Martens C., Røysland K., Goplen F.K. (2022). The Risk of Benign Paroxysmal Positional Vertigo After Head Trauma. Laryngoscope.

[17] Bhattacharyya N., Gubbels S.P., Schwartz S.R., Edlow J.A., El-Kashlan H., Fife T., Holmberg J.M., Mahoney K., Hollingsworth D.B., Roberts R. (2017). Clinical Practice Guideline: Benign Paroxysmal Positional Vertigo (Update). Otolaryngol. Head Neck Surg..

[18] Dougherty J.M., Carney M., Hohman M.H., Emmady P.D. (2023). Vestibular Dysfunction. StatPearls [Internet].

[19] Renner V., Geißler K., Boeger D., Buentzel J., Esser D., Hoffmann K., Jecker P., Mueller A., Radtke G., Axer H. (2017). Inpatient Treatment of Patients Admitted for Dizziness: A Population-Based Healthcare Research Study on Epidemiology, Diagnosis, Treatment, and Outcome. Otol. Neurotol..

[20] Guerra-Jiménez G., Arenas Rodríguez A., Falcón González J.C., Plasencia D.P., Macías Á.R. (2017). Epidemiology of vestibular disorders in the otoneurology unit. Acta Otorrinolaringol. Esp..

[21] Hanci D., Ulusoy S., Muluk N.B., Cingi C. (2015). Do viral infections have a role in benign paroxysmal positional vertigo?. B-ENT.

[22] Karabulut M., Kutlu S., Viechtbauer W., Melliti A., Meço C., Mohamad A., Özgirgin O.N., Bhandari A., Kingma H., van de Berg R. (2025). Co-occurrence of Otologic Disorders and Benign Paroxysmal Positional Vertigo: A Systematic Review and Meta-Analysis. Clin. Exp. Otorhinolaryngol..

[23] Gizzi M., Ayyagari S., Khattar V. (1998). The familial incidence of benign paroxysmal positional vertigo. Acta Otolaryngol..

[24] Dan-Goor E., Eden J.C., Wilson S.J., Dangoor J., Wilson B.R. (2010). Benign paroxysmal positional vertigo after decompression sickness: A first case report and review of the literature. Am. J. Otolaryngol..

[25] Chen G., Li Y., Si J., Zhao X., Zhang T., Dai X., Yu G. (2019). Treatment and recurrence of traumatic versus idiopathic benign paroxysmal positional vertigo: A meta-analysis. Acta Otolaryngol..

[26] Park S.K., Kim S.Y., Han K.H., Hong S.K., Kim J.S., Koo J.-W. (2013). Benign paroxysmal positional vertigo after surgical drilling of the temporal bone. Otol. Neurotol..

[27] Ahn S.K., Jeon S.Y., Kim J.P., Park J.J., Hur D.G., Kim D.-W., Woo S.-H., Kwon O.-J. (2011). Clinical characteristics and treatment of benign paroxysmal positional vertigo after traumatic brain injury. J. Trauma.

[28] Jeong S.H., Choi S.H., Kim J.Y., Koo J.W., Kim H.J., Kim J.S. (2009). Osteopenia and osteoporosis in idiopathic benign positional vertigo. Neurology.

[29] Han W., Fan Z., Zhou M., Guo X., Yan W., Lu X., Li L., Gu C., Chen C., Wu Y. (2018). Low 25-hydroxyvitamin D levels in postmenopausal female patients with benign paroxysmal positional vertigo. Acta Otolaryngol..

[30] D’Silva L.J., Staecker H., Lin J., Sykes K.J., Phadnis M.A., McMahon T.M., Connolly D., Sabus C.H., Whitney S.L., Kluding P.M. (2016). Retrospective data suggests that the higher prevalence of benign paroxysmal positional vertigo in individuals with type 2 diabetes is mediated by hypertension. J. Vestib. Res..

[31] Sreenivas V., Sima N.H., Philip S. (2021). The Role of Comorbidities in Benign Paroxysmal Positional Vertigo. Ear Nose Throat J..

[32] Bruss D., Abouzari M., Sarna B., Goshtasbi K., Lee A., Birkenbeuel J., Djalilian H.R. (2021). Migraine Features in Patients with Recurrent Benign Paroxysmal Positional Vertigo. Otol. Neurotol..

[33] Chu C.H., Liu C.J., Lin L.Y., Chen T.J., Wang S.J. (2015). Migraine is associated with an increased risk for benign paroxysmal positional vertigo: A nationwide population-based study. J. Headache Pain.

[34] Higashi-Shingai K., Imai T., Kitahara T., Uno A., Ohta Y., Horii A., Nishiike S., Kawashima T., Hasegawa T., Inohara H. (2011). Diagnosis of the subtype and affected ear of benign paroxysmal positional vertigo using a questionnaire. Acta Otolaryngol..

[35] Li L., Liu J.G., Wang Z.W., Qi X.K. (2017). Formulation and evaluationof diagnostic questionnaire for benign paroxysmal positional vertigo. Zhonghua Yi Xue Za Zhi.

[36] Kim H.J., Song J.M., Zhong L., Yang X., Kim J.S. (2020). Questionnaire-based diagnosis of benign paroxysmal positional vertigo. Neurology.

[37] Feil K., Feuerecker R., Goldschagg N., Strobl R., Brandt T., von Muller A., Grill E., Strupp M. (2018). Predictive capability of an iPad-based medical device (medx) for the diagnosis of vertigo and dizziness. Front. Neurol..

[38] Lim E.C., Park J.H., Jeon H.J., Kim H.J., Lee H.J., Song C.G., Hong S.K. (2019). Developing a diagnostic decision support system for benign paroxysmal positional vertigo using a deep-learning model. J. Clin. Med..

[39] von Brevern M., Bertholon P., Brandt T., Fife T., Imai T., Nuti D., Newman-Toker D. (2015). Benign paroxysmal positional vertigo: Diagnostic criteria. J. Vestib. Res..

[40] Halker R.B., Barrs D.M., Wellik K.E., Wingerchuk D.M., Demaerschalk B.M. (2008). Establishing a diagnosis of benign paroxysmal positional vertigo through the dix-hallpike and side-lying maneuvers: A critically appraised topic. Neurologist.

[41] Nuti D., Zee D., Bronstein A. (2013). Positional vertigo and benign paroxysmal positional vertigo. Oxford Textbook of Vertigo and Imbalance.

[42] Cox H., Frith J. (2025). Best practice assessment and management of benign paroxysmal positional vertigo in older adults. Age Ageing.

[43] Steddin S., Ing D., Brandt T. (1996). Horizontal canal benign paroxysmal positioning vertigo (h-BPPV): Transition of canalolithiasis to cupulolithiasis. Ann. Neurol..

[44] Yetiser S., Ince D. (2015). Diagnostic Role of Head-Bending and Lying-Down Tests in Lateral Canal Benign Paroxysmal Positional Vertigo. Otol. Neurotol..

[45] Choung Y., Shin Y., Kahng H., Park K., Choi S.J. (2006). «Bow and lean test» to determine the affected ear of horizontal canal benign paroxysmal positional vertigo. Laryngoscope.

[46] Arán-Tapia I., Bastos G., P Muñuzuri A. (2025). Optimization of the Yacovino maneuver for superior canal BPPV using numerical simulations. Hear Res..

[47] Bhandari A., Bhandari R., Kingma H., Strupp M. (2021). Diagnostic and Therapeutic Maneuvers for Anterior Canal BPPV Canalithiasis: Three-Dimensional Simulations. Front. Neurol..

[48] Jeon E.J., Lee D.H., Park J.M., Oh J.H., Seo J.H. (2019). The efficacy of a modified Dix-Hallpike test with a pillow under shoulders. J. Vestib. Res..

[49] Andera L., Azeredo W.J., Greene J.S., Sun H., Walter J. (2020). Optimizing Testing for BPPV—The Loaded Dix-Hallpike. J. Int. Adv. Otol..

[50] Evren C., Demirbilek N., Elbistanlı M.S., Köktürk F. (2017). Diagnostic value of repeated Dix-Hallpike and rollmaneuvers in benign paroxysmal positional vertigo. Braz. J. Otorhinolaryngol..

[51] Li W., Sun J., Zhao Z., Xu J., Wang H., Ding R., Zhang Y. (2023). Efficacy of Epley’s maneuver plus betahistine in the management of PC-BPPV: A systematic review and meta-analysis. Medicine.

[52] Anurin I., Ziemska-Gorczyca M., Pavlovschi D., Kantor I., Dżaman K. (2023). The Impact of the Angular Head Movement’s Velocity during Diagnostic Maneuvers on Proper Benign Positional Paroxysmal Vertigo Diagnosis and Therapy. Diagnostics.

[53] Lindell E., Finizia C., Davidsson H., Kollen L., Kern S., Skoog I., Rydén L. (2024). Prevalence of benign paroxysmal positional vertigo in a population-based setting among 75-year-olds. J. Vestib. Res..

[54] Covert K., Davis B., Hammonds K., O’Neil M., Mucha A. (2026). Risk Factors for Benign Paroxysmal Positional Vertigo in an Acutely Concussed Adolescent Population. J. Head Trauma Rehabil..

[55] Little C.C., Schwam Z.G., Campo M., Gurley J., Hujsak B., Cosetti M.K., Kelly J. (2022). Immediate Improvement in Subjective Visual Vertical and Disequilibrium Predicts Resolution of Benign Paroxysmal Positional Vertigo Following Single Canalith Repositioning Maneuver. Otol. Neurotol. Open.

[56] Hentze M., Hougaard D.D., Kingma H. (2024). Is diagnostics of Benign Paroxysmal Positional Vertigo with a mechanical rotation chair superior to traditional manual diagnostics? A randomized controlled crossover study. Front. Neurol..

[57] Bech M.W., Staffe A.T., Hougaard D.D. (2023). A mechanical rotation chair provides superior diagnostics of benign paroxysmal positional vertigo. Front. Neurol..

[58] Chaure-Cordero M., Garrote-Garrote M., Esteban-Sánchez J., Morales-Chacchi P., Del Valle-Díaz M., Martin-Sanz E. (2024). Comparison between Classical- and Rotational-Mechanical-Chair-Assisted Maneuvers in a Population of Patients with Benign Paroxysmal Positional Vertigo. J. Clin. Med..

[59] Hentze M., Hougaard D.D., Kingma H. (2025). Impact of head orientation and head movement in traditional manual diagnostics of benign paroxysmal positional vertigo: A randomized controlled crossover study. Front. Neurol..

[60] Chaure-Cordero M., Martin-Sanz E. (2026). Rotational Chair vs. Manual Maneuvers for Non-Complex BPPV: A Prospective Randomized Study. Laryngoscope.

[61] Norré M.E. (1994). Diagnostic problems in patients with benign paroxysmal positional vertigo. Laryngoscope.

[62] Boniver R. (2008). Benign paroxysmal positional vertigo: An overview. Int. Tinnitus J..

[63] Bronstein A.M. (2003). Benign paroxysmal positional vertigo: Some recent advances. Curr. Opin. Neurol..

[64] Ganança M.M., Caovilla H.H., Ganança F.F. (2010). Electronystagmography versus videonystagmography. Braz. J. Otorhinolaryngol..

[65] Barber H.O., Stockwell C.W. (1980). Manual of Electronystagmography.

[66] Ganança M.M., Caovilla H.H., Munhoz M.S.L., Silva M.L.G., Ganança F.F., Ganança C.F. (2009). Clinical features of benign paroxysmal positional vertigo. Braz. j. otorhinolaryngol..

[67] Houston H.G., Watson D.R. (1994). A review of computerized electronystagmography technology. Br. J. Audiol..

[68] Watanabe Y., Takeda S. (1996). Computerized electro-nystagmography. Acta Otolaryngol. Suppl..

[69] Stockwell C.W. (2004). Introduction to ENG.

[70] Barin K. (2008). Comprehensive Guide to VNG/ENG Administration and Interpretation.

[71] Eggert T. (2007). Eye movement recordings: Methods. Dev. Ophthalmol..

[72] Yildiz M.G., Bilal N., Kara I., Sagiroglu S., Orhan I., Doganer A. (2021). Characteristics of Benign Paroxysmal Positional Vertigo Following an Earthquake. Ann. Otol. Rhinol. Laryngol..

[73] Polat A.P., Demir S., Kale Ö., Kuntman B.D., Erbek H.S. (2023). Evaluation of Mental Rotation Ability in Patients with Unilateral Benign Paroxysmal Positional Vertigo. J. Int. Adv. Otol..

[74] Balatsouras D.G., Korres S.G. (2012). Subjective benign paroxysmal positional vertigo. Otolaryngol. Head Neck Surg..

[75] Bojrab D.I., Maya Kato B., Glasscock M.E., Aina J.G. (2003). Vestibular testing. Glasscock-Shambaugh Surgery of the Ear.

[76] Boniver R. (1998). Videonystagmography versus electronystagmography: Advantages and disadvantages. Neurootology Newsl..

[77] VanDerHeyden C.M., Carender W.J., Heidenreich K.D. (2015). Nystagmus discordance with 2-dimensional videonystagmography in posterior semicircular canal benign paroxysmal positional vertigo. Otolaryngol. Head Neck Surg..

[78] Halmágyi G.M., Akdal G., Welgampola M.S., Wang C. (2023). Neurological update: Neuro-otology 2023. J. Neurol..

[79] Young A.S., Lechner C., Bradshaw A.P., MacDougall H.G., Black D.A., Halmagyi G.M., Welgampola M.S. (2019). Capturing acute vertigo: A vestibular event monitor. Neurology.

[80] Liu Z., Wang Y., Zhu M., Zhang J., He B. (2025). Bppv nystagmus signals diagnosis framework based on deep learning. Phys. Eng. Sci. Med..

[81] Wu P., Liu X., Dai Q., Yu J., Zhao J., Yu F., Liu Y., Gao Y., Li H., Li W. (2023). Diagnosing the benign paroxysmal positional vertigo via 1D and deep-learning composite model. J. Neurol..

[82] Chaturvedi K., Yang N., Hannigan I., Reid N., Bradshaw A., Thiyagarajan K., Jan T., Braytee A., Welgampola M.S., Prasad M. (2026). Automated classification of benign paroxysmal positional vertigo from video-nystagmography using a delay-aware neural network. Sci. Rep..

[83] Newman J.L., Phillips J.S., Cox S.J. (2021). Detecting positional vertigo using an ensemble of 2D convolutional neural networks. BioMed Signal Process Control.

[84] Oya R., Imai T., Takenaka Y., Sato T., Oshima K., Ohta Y., Inohara H. (2019). Clinical significance of cervical and ocular vestibular evoked myogenic potentials in benign paroxysmal positional vertigo: A meta-analysis. Eur. Arch. Otorhinolaryngol..

[85] Chen G., Dai X., Ren X., Lin N., Zhang M., Du Z., Zhang E. (2020). Ocular vs. Cervical Vestibular Evoked Myogenic Potentials in Benign Paroxysmal Positional Vertigo: A Systematic Review and Meta-Analysis. Front. Neurol..

[86] Shupak A., Falah R., Kaminer M. (2020). Functional Integrity of the Inferior Vestibular Nerve and Posterior Canal BPPV. Front. Neurol..

[87] Chen G., Zhao X., Yu G., Jian H., Li Y., Xu G. (2019). Otolith dysfunction in recurrent benign paroxysmal positional vertigo after mild traumatic brain injury. Acta Otolaryngol..

[88] Cheon T.U., Park J.H., Lee J.S., Bae S.H. (2025). Defining diagnostic thresholds for dissociation between caloric test and vHIT in Ménière’s disease. Front. Neurol..

[89] Iida M., Naitoh A., Aihara H., Takahashi H., Hitouji K., Nomura K. (1998). Evaluation of vertical semicircular canal function by the caloric test--a study on patients with benign paroxysmal positional vertigo. Tokai J. Exp. Clin. Med..

[90] Bi J., Liu B., Zhang Y., Duan J., Zhou Q. (2019). Caloric tests in clinical practice in benign paroxysmal positional vertigo. Acta Otolaryngol..

[91] Elsherif M., Eldeeb D., Eldeeb M. (2021). Clinical significance of video head impulse test in benign paroxysmal positional vertigo: A meta-analysis. Eur. Arch. Otorhinolaryngol..

[92] Saltürk Z., Yetişer S. (2020). Video head impulse testing in patients with benign paroxysmal positional vertigo. Acta Otolaryngol..

[93] Abdulrahim R., Bhandary B.S.K., Rajeshwary A., Goutham M.K., Bhat V., Saldanha M. (2022). The Role of Video Head Impulse Test (Vhit) in Diagnosing Benign Paroxysmal Positional Vertigo (BPPV). Indian J. Otolaryngol. Head Neck Surg..

[94] Kabaya K., Katsumi S., Fukushima A., Esaki S., Minakata T., Iwasaki S. (2023). Assessment of semicircular canal function in benign paroxysmal positional vertigo using the video head impulse test and caloric test. Laryngoscope Investig. Otolaryngol..

[95] Molnár A., Maihoub S., Tamás L., Szirmai Á. (2022). A possible objective test to detect benign paroxysmal positional vertigo. The role of the caloric and video-head impulse tests in the diagnosis. J. Otol..

[96] Zhang Y., Wang Y., Zhen Z., Zhang J., Zeng Z., Zhong Z., Wang Q. (2023). The role of electrocochleography and the caloric test in predicting short-term recurrence of benign paroxysmal positional vertigo. Front. Neurol..

[97] Giacomini P.G., Alessandrini M., Magrini A. (2002). Long-term postural abnormalities in benign paroxysmal positional vertigo. ORL J. Otorhinolaryngol. Relat. Spec..

[98] Di Fabio R.P. (1996). Meta-analysis of the sensitivity and specificity of platform posturography. Arch. Otolaryngol. Head Neck Surg..

[99] Zamysłowska-Szmytke E., Janc M., Ławnicki K., Śliwińska-Kowalska M. (2022). Zastosowanie posturografii dla oceny układu równowagi dla potrzeb medycyny pracy [Use of the posturography in balance system evaluation in occupational medicine]. Med. Pr..

[100] Lloyd M., Mackintosh A., Grant C., McManus F., Kelly A.M., Karunajeewa H., Tang C.Y. (2020). Evidence-based management of patients with vertigo, dizziness, and imbalance at an Australian metropolitan health service: An observational study of clinical practice. Physiother. Theory Pract..

[101] Luryi A.L., LaRouere M., Babu S., Bojrab D.I., Zappia J., Sargent E.W., Schutt C.A. (2019). Traumatic versus Idiopathic Benign Positional Vertigo: Analysis of Disease, Treatment, and Outcome Characteristics. Otolaryngol. Head Neck Surg..

[102] Qian S.X., Li F., Zhuang J.H., Chen Y., Yang H.L., Zhou X.W., Gu H.H., Gao B. (2017). Misdiagnosis and associated costs of benign paroxysmal positional vertigo. Zhonghua Yi Xue Za Zhi.

[103] Arshad M., Abbas S., Qureshi I.A. (2013). Delay in diagnosis and treatment of benign paroxysmal positional vertigo in current practice. J. Ayub Med. Coll. Abbottabad.

